# Exploring Factors Associated with Recent HIV Testing among Heterosexuals at High Risk for HIV Infection Recruited with Venue-based Sampling

**DOI:** 10.4172/2155-6113.1000544

**Published:** 2016-02-15

**Authors:** Marya Gwadz, Charles M. Cleland, Samuel M. Jenness, Elizabeth Silverman, Holly Hagan, Amanda S. Ritchie, Noelle R. Leonard, Talaya McCright-Gill, Belkis Martinez, Quentin Swain, Alexandra Kutnick, Dawa Sherpa

**Affiliations:** 1 New York University College of Nursing, USA; 2 Emory University, USA; 3 The BCAP Collaborative Research Team, USA

**Keywords:** Venue-based sampling, HIV testing, Individual barriers, Social barriers, Structural barriers, Heterosexuals, Seek test treat and retain, Sex difference, Access

## Abstract

Annual HIV testing is recommended for high-risk populations in the United States, to identify HIV infections early and provide timely linkage to treatment. However, heterosexuals at high risk for HIV, due to their residence in urban areas of high poverty and elevated HIV prevalence, test for HIV less frequently than other risk groups, and late diagnosis of HIV is common. Yet the factors impeding HIV testing in this group, which is predominantly African American/Black and Latino/Hispanic, are poorly understood. The present study addresses this gap. Using a systematic community-based sampling method, venue-based sampling (VBS), we estimate rates of lifetime and recent (past year) HIV testing among high-risk heterosexuals (HRH), and explore a set of putative multi-level barriers to and facilitators of recent testing, by gender. Participants were 338 HRH African American/Black and Latino/Hispanic adults recruited using VBS, who completed a computerized structured assessment battery guided by the Theory of Triadic Influence, comprised of reliable/valid measures on socio-demographic characteristics, HIV testing history, and multi-level barriers to HIV testing. Logistic regression analysis was used to identify factors associated with HIV testing within the past year. Most HRH had tested at least once (94%), and more than half had tested within the past year (58%), but only 37% tested annually. In both men and women, the odds of recent testing were similar and associated with structural factors (better access to testing) and sexually transmitted infection (STI) testing and diagnosis. Thus VBS identified serious gaps in rates of annual HIV testing among HRH. Improvements in access to high-quality HIV testing and leveraging of STI testing are needed to increase the proportion of HRH testing annually for HIV. Such improvements could increase early detection of HIV, improve the long-term health of individuals, and reduce HIV transmission by increasing rates of viral suppression.

## Introduction

Of the 1.2 million individuals living with HIV in the United States, 14% are unaware of their diagnoses [1]. Yet between 44–66% of new HIV infections each year are attributed to this small proportion of persons living with undiagnosed HIV [2]. Since 2006, the Centers for Disease Control and Prevention (CDC) has recommend at least annual testing for persons at high risk for HIV as part of the national HIV prevention strategy [3]. Although rates of lifetime HIV testing are increasing, the most recent estimates indicate less than half of the U.S. population (44%) has ever been tested. History of testing is most common among African Americans/Blacks (63.9%), followed by non-Hispanic Whites (42.9%) and then Mexican Americans (35.7%) [4]. Yet African Americans/Blacks and Hispanics/Latinos continue to be disproportionately affected by HIV [1]. For example, African Americans/Blacks are more likely than Whites to be diagnosed late in the course of their HIV disease [5], to be diagnosed with AIDS at the time of or shortly after being diagnosed with HIV [6], and to not have a previous negative HIV test before their HIV diagnosis [7]. These racial/ethnic disparities are driven in part by insufficient rates of regular and repeated HIV testing. In fact, a recent national study of high-risk populations indicated that fewer than half of vulnerable African Americans/Blacks and Hispanics/Latinos had been tested in the past year [8]. Thus increasing the proportion of African Americans/Blacks and Hispanics/Latinos who have been tested, as well improving as the regularity of HIV testing, are critical to reducing racial/ethnic disparities in HIV.

Another challenge for scaling-up HIV testing is a lack of focus on high-risk heterosexuals. HIV testing is less common among heterosexuals than other risk groups such as men who have sex with men and persons who inject drugs [4]. For example, in New York City, only 31% of high-risk heterosexual men and 35% of women had recently tested despite nearly all (>90%) encountering settings where HIV testing was offered [9]. Yet heterosexuals make up a significant proportion of new HIV infections (27% nationally; [10], and account for the second highest percentage of those living with undiagnosed HIV infection (17%) [11]. However the factors influencing testing rates among high-risk heterosexuals (HRH) have not been studied as extensively as in other risk groups [12,13]. In part, this research has been hampered by the lack of an accepted definition of the population [14]. The present study uses the definition of HRH developed for the first cycle of the National HIV Behavioral Surveillance (NHBS) system conducted by the Centers for Disease Control and Prevention [15]. Grounded in this NHBS study, we define heterosexuals at high-risk for HIV as persons linked to urban geographical areas with high rates of both poverty and reported cases of heterosexually transmitted HIV [16]. African American/Black and Hispanic/Latino populations comprise the majority of the population in these “high-risk areas” (HRAs) [17].

In response to the insufficient rates of lifetime, as well as annual, HIV testing among HRH, the Centers for Disease Control and Prevention has called for research to better identify and overcome testing barriers in this group [18]. The present study addresses this gap using a rigorous sampling method to access HRH directly in their communities; namely, venue-based sampling (VBS). VBS is designed to systematically recruit individuals within a target population who may be hidden from standard sampling approaches (e.g., household-based sampling or convenience sampling in community-based organizations), but who may be accessible in identifiable public venues such as parks, churches, and hair salons. VBS has proven successful in identifying populations at high risk of HIV infection, mainly men who have sex with men [19–21], but also some studies of heterosexuals in the US and globally [22–24].

The present study explores a set of putative barriers to and facilitators of past-year HIV testing among African American/Black and Hispanic/Latino HRH. These factors are drawn from the empirical literature and conceptualized in the framework of the Theory of Triadic Influence [25], a social-cognitive theory delineating three “streams of influence” on health behavior: the individual/attitudinal-, social-, and structural-levels of influence. The theoretical model is described in more detail elsewhere [26] and summarized briefly here. Individual/attitudinal barriers to regular HIV testing among HRH may include mistrust of medical environments and “competing priorities” such as substance use [27,28], as well as unemployment, and unstable housing, all of which are complicated by low socioeconomic status [29,30]. At the social level of influence, unfavorable peer norms regarding testing can serve as deterrents [31]. At the structural level, HRH often have insufficient access to settings where high-quality HIV testing is offered [9,32]. Concurrently, facilitators of testing operate among HRH, such as intrinsic motivation to achieve good health, and involvement in health care and other settings that provide needed services [9,33]. Importantly, we would expect patterns of barriers to/facilitators of HIV testing to differ by gender, driven by factors such as a greater likelihood of childcare responsibilities among women [34], and gender differences in access to health care settings where HIV testing is offered, such as settings that provide gynecological and prenatal care available to women, and higher incarceration rates among men [9].

The present study’s aims, therefore, are to provide a description of our novel VBS sampling design, in which we used administrative data to define and enumerate potential recruitment locations to reach and engage HRH; to estimate rates of lifetime and recent (past year) testing among the sampled cohort of HRH; and explore multi-level barriers to/facilitators of recent testing, by gender.

## Methods

### Sampling and recruitment

Study participants (N=338) were recruited in 2012–2015 in New York City using VBS as part of a larger study on undiagnosed HIV infection among HRH – a “Seek, Test, Treat, and Retain” (STTR) study [26]. VBS starts by identifying days and times at which the target population gathers at specific venues, constructing a sampling frame of venue-day-time units (VDTs), randomly selecting and visiting VDTs (the primary sampling units), and systematically intercepting and collecting information from consenting members of the target population [19]. The VBS protocol used in the present study has been described in detail elsewhere [26], and is described in brief below. The study was approved by the Institutional Review Board of the New York University Langone School of Medicine.

### Setting

**Figure 1:**
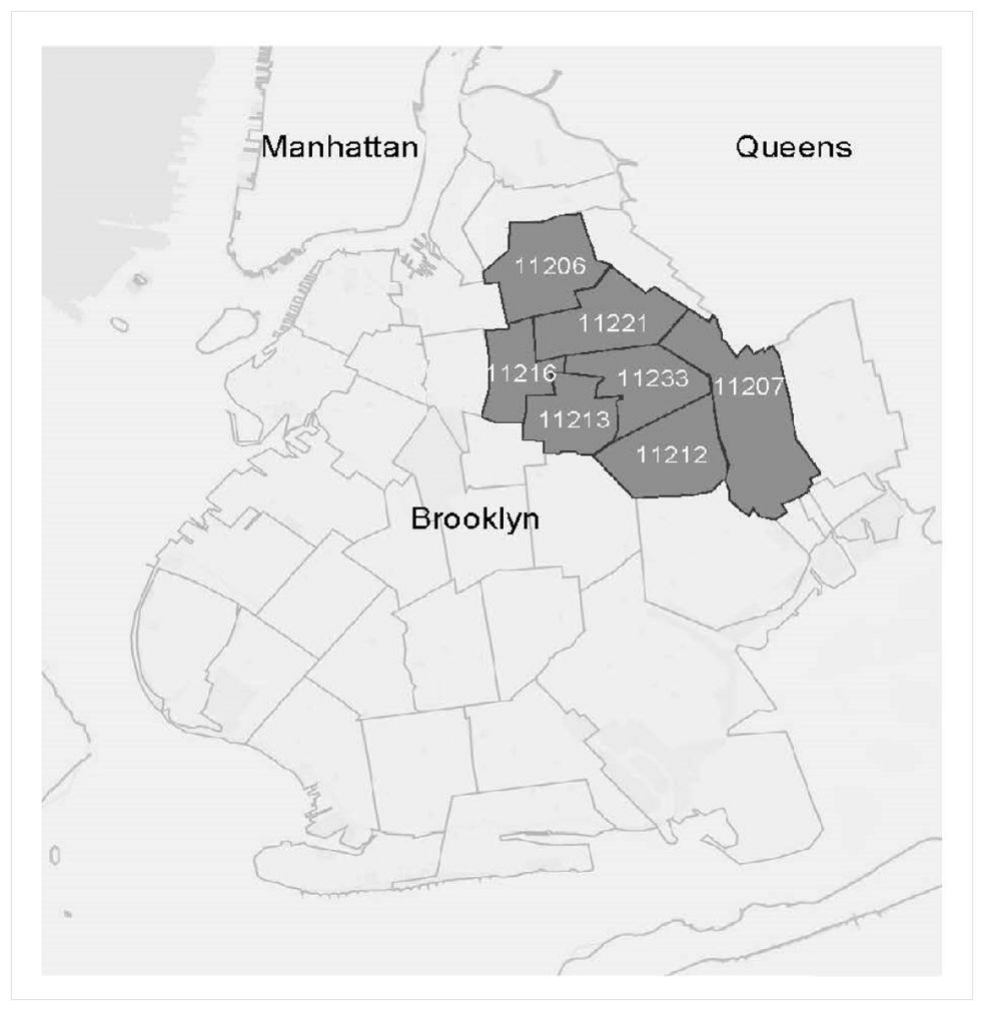
Core high-risk area (HRA) in the borough of Brooklyn.

The study was located in a well-defined HRA in Brooklyn, the borough (out of five boroughs) in New York City with the highest local heterosexual HIV prevalence. The HRA was defined at the inception of the study by rank ordering all postal zip codes in Brooklyn based on levels of heterosexual HIV prevalence and household poverty. A core HRA was then selected from that listing that comprised of the top 25% of zip codes on the HRA index (seven contiguous zip codes in total; (Figure 1) [26].

Within the full set of the seven Brooklyn HRA zip codes, we used a novel approach to define specific social venues. The core concept of VBS is that the target population congregates at definable and identifiable social venues or spaces. Because spaces where HRH congregate may be virtually unlimited in number or scope, particularly compared to other VBS target populations such as men who have sex with men who may be more concentrated, our study used administrative data to define and enumerate HRH venues for the purpose of our VBS sampling. We assumed HRH congregation would be associated with the abundance and clustering of business/commercial space in the community. First, we used New York City Department of Urban Planning Data from 2009 that contained a listing of each building within all HRA zip codes for Brooklyn. These data contained information on the building size and zoning (residential, commercial, or mixed) for each land parcel that was nested within census blocks. For each census block, we calculated the sum of the commercial space within that block. Sampling “venues” were defined as blocks at the upper 80% quantile of blocks on this commercial space metric within the HRA. To verify this specific quantile, we used mixed quantitative (enumerating pedestrians visible in Google “street view” data) and qualitative (in-person ethnographic reports of a sample of blocks) approaches. In addition to these commercial venues, we also added discrete venues for two other core categories: parks, playgrounds, and related green spaces; and public housing projects.

#### Study eligibility criteria were

Age 18–60 years; sexually active (vaginal and/or anal sex) with at least one opposite-sex partner in the previous year; residence in the core HRA; African American/Black or Hispanic/Latino race/ethnicity; comprehension of English or Spanish; unknown or negative HIV status; and not actively psychotic [35,36].

### Design

#### Enrollment and baseline interview

Up to three VDTs were selected for participant enrollment events each month, with the goal of recruiting up to nine participants per event. At each event, a pre-specified “recruitment line” in the selected venue was demarcated. For commercial block venues, this consisted of the study team choosing the most appropriate sidewalk space on the randomly sampled block. Individuals who crossed that line were approached by study staff, pre-screened for age and whether they resided in the HRA, and then asked if they would be willing to participate in a brief health screening interview for a “community health study.” Those found eligible for the study were then brought to a confidential location, where the study was explained to them. Those interested provided signed informed consent and participated in a baseline interview using Audio Computer-Assisted Self-Interviewing. Participants received compensation of $15 for the screening interview and $30 for the baseline interview. A total of 23,795 potential participants were identified in the venues in 60 recruitment events over 30 months (an average of 396.58 individuals identified per event). Of these, a total of 3,183 were approached on the street (an average of 53.05 individuals approached per event), and 880 (27.7%, 880/3183) were found potentially eligible for the study based on pre-screening for age and residence in the HRA. A total of 565 were screened for eligibility (64.2% of those potentially eligible, 565/880) and completed the screening interview, and of these, 428 (75.8%, 428/565) were found to be eligible, and 403 (94.2%, 403/428) were enrolled and completed the baseline assessment. Of these 403 enrolled participants, 40 are not included in the present study, because they were enrolled in the study’s initial phase, which explicitly excluded those who had been tested in the past year. This criterion was later changed, as described elsewhere [26]. Four participants were excluded due to missing data on one or more potential predictors of HIV testing in the past year. Further, 21 participants could not provide the date of their last HIV test, and these cases were classified as missing for past-year testing and excluded from analysis, a conservative approach. Thus, 338 participants (84% of enrolled) were included in the analysis.

### Measures

The domains assessed in the present study are drawn from the theoretical model described above, and also include socio-demographic, background, and risk factors for HIV and other poor health outcomes. The measures used in the present study were drawn primarily from a set of “harmonized” instruments used for the Seek, Test, Treat, and Retain (STTR) projects sponsored by the National Institute on Drug Abuses (NIDA) at the National Institutes of Health [37]. These measures are reliable and valid, have been used in past studies with HRH and similar vulnerable populations, and are described in brief below.

Age, sex, race/ethnicity, sexual orientation, marital status, children, education, insurance and housing status, employment, financial insecurity, and history of incarceration were measured using a structured NIDA-harmonized instrument [38,39]. We assessed testing for sexually transmitted infections (STIs) and STI diagnoses recently and over the lifetime [40]; depression over the past week (20-item CES-D; α=0.80); and current anxiety (6-item BSI anxiety; α=0.88). Composite depression and anxiety scores were calculated and cut-offs of 16 or greater and 0.7 or greater, respectively, used to determine presence or absence of symptoms at a clinically significant level [41,42]. A measure developed by the NHBS system was used to assess lifetime and past month same-sex and heterosexual partners, experiences of group and unprotected sex, and lifetime experiences of exchanging of sex for money or drugs [43]. The frequency of tobacco, drug, and alcohol use in the past month [44]; lifetime and past month history of injection drug use [40]; drug problems in the past year (TCU Drug Screen; 9 items; Cronbach’s alpha (α)=0.91) [45]; and alcohol problems in the past year (AUDIT; 10 items; α=0.89) [46] were assessed using validated measures. “Problem” drug and alcohol use were coded using established criteria [44,45].

We assessed mistrust of the medical system via HIV conspiracy beliefs, for example, the belief that the government is withholding a cure for HIV (5 items rated on a 5-point Likert-type scale; α=0.70); items were scored from 0 (strongly disagree) to 4 (strongly agree) and total mistrust was the average across items [47]. We assessed perceptions of peer norms regarding HIV testing (7 items rated on a 7-point Likert-type scale; α=0.59) e.g., “how many of your close friends or family are afraid to get an HIV test?”; items were scored from 0 (none) to 6 (all) and the total was the average across items [48]. We assessed perceived ease of access to HIV testing facilities (14 items rated on a 5-point Likert-type scale; α=0.81); items were scored from 0 (strongly disagree) to 4 (strongly agree) and total access was the average across items [49]. Lifetime and past-year HIV testing were assessed based on self-report [50].

### Data Analysis

Logistic regression was used to estimate bivariate associations between predictors and HIV testing in the past year separately for women and men. Logistic regression was also used to estimate associations in multivariable models separately for women and men. Multivariable models started with main effects of 26 potential predictors of HIV testing in the past year. Starting with terms furthest from significance, terms were removed if the associated p-value (p) was greater than 0.10. This backward elimination of non-significant terms used the method of Lawless and Singhal [51] implemented in the rms package [52] of the R statistical computing environment [53], which was used for all analyses. For bivariate associations and in final multivariable models, tests of statistical significance were two-tailed, and p<0.05 was considered significant.

## Results

### Participant characteristics

Table 1 shows socio-demographic and background factors, sexual behavior, drug and alcohol use and problems, sexually transmitted infection history, individual-attitudinal factors, social-level factors, and structural-level factors potentially related to testing for HIV in the past year. About 55% of participants were men, and most (74%) were non-Hispanic African Americans/Blacks. Age ranged from 18 to 60 years, with a mean age of 34 years (SD=12 years). Many (35%) had not completed high school or attained a GED, and more than one-third (36%) had experienced homelessness. More than half (51%) had been incarcerated, 46% of those within the past year. More than half (54%) had sex without a condom in the past month, and 22% had more than one sex partner in the past month. Although most (83%) had health insurance, many (75%) reported they ran out of money for basic necessities in the past year.

Most had tested for HIV at least once prior to the study (94%), and more than half reported testing for HIV within the past year (58%). To assess regularity of HIV testing since CDC’s annual testing recommendation began in 2006, we calculated the ratio of number of lifetime HIV tests to number of years after the recommendation began in which the participant was at least eighteen years of age [3]. A ratio of 1 would indicate adherence to annual testing during years of adulthood, on average. Our observed ratio ranged from zero to twenty, with a median of 0.73. Only a minority of participants (37%) reported as many HIV tests as the number of adult years since the annual testing recommendation began in 2006, indicating consistent annual testing was not common.

### Predictors of HIV Testing in the Past Year

Table 2 shows bivariate associations between testing for HIV in the past year and other variables, by gender. Among women, older age (odds ratio [OR]=0.55) and being married (OR=0.44) were associated with a decrease in the odds of testing for HIV in the past year while better access to HIV testing (OR=1.70) was associated with an increase in those odds (*p* < 0.05). Among men, a heterosexual orientation (some participants identified as bisexual; OR=4.69), lifetime STI testing (OR=2.44), and better access to HIV testing (OR=1.71) were associated with an increase in the odds of testing for HIV in the past year (*p* < 0.05).

Table 3 shows adjusted associations between testing for HIV in the past year and other variables. Among women, only older age (AOR=0.55) was associated with a decrease in the odds of testing for HIV in the past year. Among men, only better access to HIV testing (AOR=1.71) was associated with an increase in the odds of testing for HIV in the past year. When women and men were included together in one model, better access to HIV testing (AOR=2.59), STI testing (AOR=1.92) and STI diagnosis (AOR=2.12) were each associated with an increase in the odds of testing for HIV in the past year.

## Discussion

The present study highlights progress made in the effort to achieve high rates of HIV testing among populations at risk, as well as gaps that remain. Further, it advances what is known about the frequency of testing, and factors that facilitate testing, in an under-studied population, HRH, using a rigorous community-based sampling method, VBS, in a high-poverty area. Indeed, participants in the sample evidenced substantial vulnerabilities in a number of respects, showing overall low rates of employment, severe economic strains, and substantial rates of past incarceration. On the other hand, many were in serious partnerships/relationships, most had health insurance, and the majority was stably housed. Almost all had been tested for HIV in their lifetimes, but regular, annual testing throughout adulthood was uncommon, and only about half had been tested for HIV in the past year. While the present study does not disaggregate testing rates by race/ethnicity, CDC data indicate that 65% of African Americans/Blacks and 46% of Latinos/Hispanics have ever been tested for HIV, and rates of lifetime testing in this sample are higher than these national estimates [1]. (The CDC does not provide data on rates of annual testing; they are likely significantly lower than these). These higher observed rates of lifetime and, we estimate, recent HIV testing in this sample compared to national estimates may reflect recent trends in HIV testing, where testing frequency is increasing, and/or the local context, where the Department of Health requires an HIV test to be offered in every medical encounter [3].

Yet about half of the HRH in the sample had not been tested for HIV in the past year. Contrary to expectations, there were no substantial gender differences in the types of factors that promote or impede recent HIV testing. Instead, recent HIV testing was associated with structural factors, namely, the ease of accessing HIV testing. This suggests expanding access and improving easy access to settings where high-quality HIV testing is offered may improve annual HIV testing rates [9]. Bowleg, for example, has highlighted the barriers that African American/Black heterosexual men in particular experience to HIV testing, and recommend four strategies to improve their access to HIV prevention services, including HIV testing. These include creating men’s health programs, increasing workforce and post-incarceration release programs, forming linkages to women’s prevention programs, and developing faith-based initiatives [54]. Some of these four approaches could be applied to boost testing rates in women, and both genders may benefit from social marketing campaigns [55,56] and access to HIV self-testing [57].

Receiving testing for STIs, and receiving an STI diagnosis, were associated with recent HIV testing for males and females. STIs, with perhaps the exception of HSV-2, are generally less stigmatized than HIV, which may play a role in promoting STI testing among HRH [58]. Then, this moment of heightened sexual risk or perceived sexual risk may motivate the offer and/or acceptance of HIV testing in the medical setting [59]. Thus concerns about STIs and STI testing can play important roles in achieving the goal of HIV elimination. Yet the STI and HIV prevention systems are largely “siloed,” which may lead to missed opportunities for comprehensive sexual health services that consider both STIs and HIV infection [60,61]. The CDC has called for better integration of STI and HIV services to improve the early diagnosis of a range of sexually transmitted health problems [62]. It is also possible, given the cross-sectional nature of the data in the present study, that HIV testing triggered STI testing [63].

## Limitations

This study is exploratory in nature, and estimation of the associations between the predictor variables and the HIV testing outcomes may be influenced by type-I error due to the multiple comparisons within the model specification. Despite our using 26 predictors in the starting model, additional unmeasured variables may be associated with testing in this population. The data are based on the quality of the VBS scheme described above; this may have missed out on key subpopulations of HRH that were not present or had a small probability of being sampled based on our venue definitions. This includes high-risk persons who did not congregate around commercial businesses or parks, or were highly transient around the neighborhood. Finally, there may have been issues related to recall and social desirability biases in the reporting of HIV testing.

## Implications

The present study advances what is known about the utility of VBS for HRH, documents the persistence of gaps in annual HIV testing rates in a population at grave risk for HIV infection, and points the way to approaches to increase testing rates in this large, high-priority population.

## Figures and Tables

**Table 1:** Participant characteristics (n=338).

	Mean / %	SD
**HIV testing**		
HIV Test - lifetime	94.36	
HIV Test in the Past Year	57.99	
**Sociodemographic and background factors**		
Male Gender	54.73	
Age	33.79	11.61
African American/Black	73.37	
Latino/Hispanic	23.96	
Married, living as married	20.71	
In a long-term relationship	31.66	
Has any children	59.17	
Identifies as heterosexual	88.76	
No High School Diploma	35.21	
Completed HS or GED But No College	36.39	
Employed full or part-time	38.17	
Ran out of money for basic necessities past 12 months	74.85	
Any health insurance	83.14	
Ever homeless	36.39	
Currently homeless	11.83	
Ever incarcerated	50.89	
Past year incarceration if ever incarcerated	45.93	
**Sexual behavior**		
Lifetime Same Sex Partner(s)	13.31	
Number of sex partners past month	1.25	1.78
Sex without a condom past month	54.44	
**Drug and alcohol use and problems**		
Any Drug Use in the Past Month	30.47	
Drug Use Frequency Past Month (0–8)	1.43	2.55
Daily cigarette smoking in the past month	39.64	
Ever injected drugs not for a medical reason	6.51	
Injected drugs in the past 30 days	2.07	
**Sexually transmitted infections (STIs)**		
STI Testing Lifetime	71.60	
STI Diagnosis Lifetime	23.37	
**Individual/attitudinal-level factors**		
HIV Conspiracy Beliefs (0–4)	1.54	0.82
**Social-level factors**		
Peer norms in support of HIV testing (0–6)	4.59	0.85
**Structural-level factors**		
HIV Testing Access (0–4)	3.44	0.56

**Table 2:** Factors associated with recent HIV testing among female and male heterosexuals at high risk in New York City (n=338).

	Female (n=153)		Male (n=185)		Female Bivariate Odds Ratio		Male Bivariate Odds Ratio	
	No Recent HIV Test (n=56)	HIV Test in Past 12 Months (n=97)	No Recent HIV Test (n=86)	HIV Test in Past 12 Months (n=99)				
	Mean (SD) or %	Mean (SD) or %	Mean (SD) or %	Mean (SD) or %				
*Socio-demographic and background factors*								
Age†	38.59 (11.94)	31.62 (10.90)	32.27 (11.99)	34.54 (11.04)	0.55	***	1.22	
African American/Black	82.14	73.20	66.28	74.75	0.59		1.51	
Latino/Hispanic	17.86	23.71	30.23	22.22	1.43		0.66	
Married, living as married	35.71	19.59	17.44	16.16	0.44	*	0.91	
In a long-term relationship	46.43	35.05	26.74	24.24	0.62		0.88	
Has any children	69.64	64.95	52.33	53.54	0.81		1.05	
Heterosexual	83.93	84.54	87.21	96.97	1.05		4.69	*
No High School Diploma	35.71	29.90	37.21	38.38	0.77		1.05	
Completed HS or GED But No College	28.57	31.96	47.67	35.35	1.17		0.60	
Employed full or part-time	35.71	38.14	44.19	34.34	1.11		0.66	
Ran out of money for basic necessities past 12 months	75.00	80.41	69.77	73.74	1.37		1.22	
Any health insurance	89.29	90.72	76.74	77.78	1.17		1.06	
Ever homeless	35.71	34.02	37.21	38.38	0.93		1.05	
Currently homeless	3.57	11.34	13.95	15.15	3.45		1.10	
Ever incarcerated	30.36	35.05	66.28	64.65	1.24		0.93	
Past year incarceration if ever incarcerated	41.18	23.53	49.12	56.25	0.44		1.33	
*Sexual behavior*								
Lifetime Same Sex Partner(s)	19.64	26.80	4.65	4.04	1.50		0.86	
Lifetime Group Sex	8.93	3.09	16.28	17.17	0.33		1.07	
Number of sex partners past month†	1.09 (1.08)	1.03 (1.31)	1.22 (1.55)	1.58 (2.51)	0.93		1.17	
Sex without a condom past month	66.07	53.61	45.35	56.57	0.59		1.57	
*Drug and alcohol use and problems*								
Any Drug Use in the Past Month	17.86	23.71	37.21	38.38	1.43		1.05	
Drug Use Frequency Past Month (0-8)†	0.89 (2.25)	1.15 (2.45)	1.80 (2.70)	1.69 (2.63)	1.13		0.96	
Daily cigarette smoking in the past month	41.07	39.18	36.05	42.42	0.92		1.31	
Ever injected drugs not for a medical reason	1.79	1.03	8.14	13.13	0.57		1.71	
Injected drugs in the past 30 days	0.00	1.03	1.16	5.05	1.00		4.52	
*Sexually transmitted infections*								
STI Testing Lifetime	75.00	83.51	53.49	73.74	1.69		2.44	**
STI Diagnosis Lifetime	30.36	26.80	15.12	23.23	0.84		1.70	
*Individual/attitudinal-level factors*								
HIV Conspiracy Beliefs (0–4)†	1.50 (0.98)	1.32 (0.67)	1.69 (0.83)	1.65 (0.80)	0.79		0.95	
*Social-level factors*								
Peer norms about HIV testing (0–6)†	4.57 (0.89)	4.70 (0.85)	4.47 (0.90)	4.60 (0.77)	1.16		1.17	
*Structural-level factors*								
HIV Testing Access (0–4)†	3.44 (0.54)	3.65 (0.40)	3.16 (0.70)	3.48 (0.46)	1.70	*	1.71	***

† Odds ratios for these variables reflect the expected change in odds of recent testing for a one standard deviation increase in the variable

* p < 0.05

** p < 0.01

*** p < 0.001

**Table 3:** Factors associated with recent HIV testing among female and male heterosexuals at high risk in New York City: Multivariate Logistic Regression.

		Female			Male			Total	
	AOR	95% CI	*p* value	AOR	95% CI	*p* value	AOR	95% CI	*p* value
Age†	0.55	0.33 – 0.77	0.001						
HIV Testing Access†				1.71	1.28 – 2.35	0.001	2.59	1.69 – 4.08	<.001
STI Testing									
Never Diagnosed vs. Never Tested							1.92	1.05 – 3.69	0.035
Diagnosed vs. Never Tested							2.12	1.25 – 3.63	0.006

† Adjusted odds ratios for these variables reflect the expected change in odds of recent testing for a one standard deviation increase in the variable.
